# Effects of gold and green kiwifruit juices on the physicochemical properties and tenderness of pork loin and antioxidant activity during incubation (24 h) in a pork model system

**DOI:** 10.5713/ab.23.0410

**Published:** 2024-04-01

**Authors:** Haeun Kim, Koo Bok Chin

**Affiliations:** 1 Department of Animal Science, Chonnam National University, Gwangju 61186, Korea

**Keywords:** Antioxidant Activity, Bioactive Peptides, Hydrolysates, Incubation Time, Kiwifruit

## Abstract

**Objective:**

Although pork loins is not a tough meat, they need to develop meat products with a soft texture for the elderly. This study focused on the physicochemical properties and tenderness characteristics of pork loin injected with green kiwifruit juice (GRJ) and gold kiwifruit juice (GOJ) during various incubation times. In addition, the antioxidant activities of hydrolysate derived from the hydrolysis of pork loin by kiwifruit juice protease were evaluated.

**Methods:**

The pork loin was injected with 10% and 20% GRJ and GOJ, under various incubation times (0, 4, 8, and 24 h). Then, the physicochemical properties and tenderness of pork loins were measured. 2,2- diphenyl-1-picrylhydrazyl radical scavenging activity and reducing power were conducted to determine hydrolysate’s antioxidant activities derived from pork loin’s hydrolysis by kiwifruit juice protease.

**Results:**

GRJ had greater tenderizing ability than GOJ, even at the 10% addition. When kiwifruit juice was injected into pork loin, the tenderness increased with increasing incubation time. This was confirmed by the decrease in intensity of the myosin heavy chain (MHC) band in sodium dodecyl sulfate–polyacrylamide gel electrophoresis. In particular, the MHC band decreased at 8 h for both 10% GRJ and 20% GOJ and at 4 h for 20% GRJ alone. The highest myofibril fragmentation index and peptide solubility were observed in pork loin treated with 20% GRJ compared to the other treatments during incubation. The 10% GRJ and 20% GOJ treatments showed similar levels of antioxidant activity of the protein hydrolysates in pork loin, and 20% GRJ showed the highest activity among the treatments.

**Conclusion:**

Kiwifruit juice had protease activity, and GRJ was more useful for tenderizing meat products than GOJ. Thus, GRJ at 10% could be a potential agent to tenderize and enrich the natural antioxidant activity through the proteolysis of pork loin.

## INTRODUCTION

Industrial proteases are mainly derived from animal and microbial sources, and thus their use is often restricted due to safety and some religious issues related to food adulteration and authenticity [1]. For this reason, interest in plant-derived proteases is increasing, and studies to apply them to various meat products have been reported [2,3]. Although plant proteases are commercially available and relatively inexpensive, they can deteriorate meat product quality because of their non-uniform or over-tenderizing activity when applied to meat products without caution [4]. In order to effectively apply plant proteases in the meat products, it is necessary to precisely study the progress of tenderness of meat products as affected by the addition level and application time of plant-derived proteases.

The main proteolytic enzyme in soluble proteins of kiwifruit is actinidin in its inactive form. Actinidin, a cysteine protease, exhibits protease activity at different pH ranges, depending on the substrates. The actinidin activity on meat protein was characterized by hydrolysis of actin (a globular protein) in the pH range of 3.0 to 4.5, whereas that of myosin (a fibrillar protein) was more broadly in the pH range of 3.0 to 8.0 [5]. It is also reported that actinidin hydrolyzes a broad range of myofibrillar proteins in meat at a slower rate rather than other plant proteases [6]. Because of the variability in the actinidin content and protease activity by different type of kiwifruit and the cultivation area, it is necessary to compare the hydrolysis and quality changes induced by protein hydrolysate from different kiwifruits when applied to meat products [7].

The use of bioactive peptides in the manufacture of functional foods and nutraceuticals is receiving much interest due to their health benefits for human. The functional biopeptides, such as antioxidant activity and ACE-inhibitory of protein hydrolysates, could be produced due to the cooperation between amino acid composition and among various amino acids [8]. Zhang et al [9] reported that when five types of plant-derived proteins were hydrolyzed with actinidin, a kiwifruit protease, the ACE-inhibitory rate was 71.1% to 88.3%. Meat protein contains a high amount of essential amino acids and is also a source of bioactive peptides [8]. Saiga et al [10] hydrolyzed pork myofibrillar protein with papain, one of the cysteine proteases, and identified various antioxidant peptides. Previous research reported that applying enzymes from kiwifruit juice to meat can improve the gastric digestibility of meat proteins by increasing their high digestibility, allowing the peptides to be absorbed into the body faster than when consumed by the whole proteins [7,11]. However, few studies have evaluated the antioxidant activity and fractionation of meat protein hydrolysates obtained by applying kiwifruit juice, which has an actinidin protease.

The purpose of this study is to characterize the protease activities of green and gold kiwifruit juices (GRJ and GOJ) and compare the physicochemical properties and tenderness of pork loin injected with the juices at different addition levels (10% and 20%) during 24 h incubation. In addition, the antioxidant activity of hydrolysate the meat protein hydrolysates produced by the action of the kiwifruit enzyme was evaluated.

## MATERIALS AND METHODS

### Preparation of kiwifruit juice and measurement of protease activity

Gold kiwifruit (*Actinidia chinensis* cultivar Zesy002, marketed as SunGold) and green kiwifruit (*Actinidia deliciosa* cultivar Hayward) were used in this study. After washing, the 10 g of peeled kiwifruit was ground in a blender for 1 min and then centrifuged at 10,000×*g* for 15 min. The supernatant was taken as the kiwifruit juice and used for the experiment.

Protease activity was determined according to the method of Rawdkuen et al [2]. To measure the protease activity of GOJ and GRJ, each juice was diluted 1:1 with pH 6.8 phosphate buffer, then 10 mL was taken and mixed with 90 mL of double-distilled (dd) water. Diluted GOJ and GRJ were measured for protease activity using casein as a substrate. One unit of protease activity was defined as the amount of enzyme required to liberate 1 μmol of tyrosine in 20 min at 37°C.

### Preparation of pork loin injected with kiwifruit juice

Fresh pork loins (24 h postmortem after slaughter, 98.0±7.07 kg carcass weight) were purchased from a local meat market in Gwangju, South Korea. Pork loins were cut into equal weights (approximately 200 g) and thicknesses (6 cm) after trimming excess connective tissues and fat. Then, 10% of the weight of the pork loin was injected with brine solution using a meat injector (Turkey Injectors; Newtech, Daegu, Korea). The composition of the brine solution for each treatment is shown in Table 1. After injecting the brine solution, the samples were vacuum-tumbled for 1 h. Each injected pork loin was placed in a wrap-packaging bag and incubated at 8°C for various incubation times (0, 4, 8, and 24 h), followed by measurement of their physicochemical and textural properties.

### pH and color values (L*, a*, b*)

The pH values of pork loin samples were analyzed using a pH meter (MP120; Mettler-Toledo GmbH, Schwarzenbach, Switzerland). Standard buffer solutions at pH 4.0 and 7.0 were used to calibrate the pH meter before measurements.

A CR-10 Minolta chromameter (Minolta Ltd., Tokyo, Japan) was used to determine the color of the cross-section of the pork loin samples at six random locations. Commission Internationale de l’Eclairage (CIE) L* (lightness), a* (redness), and b* (yellowness) color coordinates were recorded under illuminant D65 and 10° standard observer. Before measurements, the colorimeter was calibrated against a white calibration tile (L* = 95.6, a* = −0.79, b* = 0.85).

### Cooking loss

Cooking loss (CL) was measured according to the method of Rakasivi and Chin [12]. The pork loin sample was heated at 75°C for 30 min and placed in a plastic bag (nylon/polyethylene pouches; 20 cm×30 cm). Each sample was weighed before and after cooking to determine the CL. Differences were calculated, and the results were expressed as percentages.

### Warner–Bratzler shear force value

The Warner–Bratzler shear force (WBSF) was measured using the method of Wheeler et al [13]. The cooked pork loin samples measuring 1.5 cm in width and 2 cm in length were cut along the direction of the muscle fiber. Each sample was measured at least 10 times by an Instron Universal Testing Machine (Model 3344; Canton, MA, USA) equipped with a Warner–Bratzler blade operating with a load cell of 500 N and crosshead speed of 50 mm/min. The maximum value was described as a shear force (kgf).

### Trichloroacetic acid-soluble peptides

The trichloroacetic acid (TCA)-soluble peptide content of the supernatant was determined by the Lowry method, as described by Rawdkuen et al [2]. Results were expressed as micromoles of tyrosine per gram of sample weight.

### Myofibril fragmentation index

The myofibril fragmentation index (MFI) was carried out using the method described by Culler et al [14]. The protein concentration was measured using the biuret method. The protein concentration of each sample was diluted to 0.5 mg/mL and calculated by multiplying the absorbance value measured at 540 nm by 200.

### Sodium dodecyl sulfate–polyacrylamide gel electrophoresis

Sodium dodecyl sulfate–polyacrylamide gel electrophoresis (SDS–PAGE) was carried out on a 12% polyacrylamide resolving gel with a 4% acrylamide stacking gel. After loading with 20 μg of sample per lane, and a Prestained SDS-PAGE Standard, broad range (Cat. #161-0318; Bio-Rad, Hercules, CA, USA), the gels were run in a Mini-PROTEAN Tetra Cell (Bio-Rad, USA) at 140 V. Gels were stained for 60 min (Coomassie Brilliant Blue R-250; Bio-Rad, USA) and destained overnight.

### Scanning electron microscopy

The structures and morphologies of the meat samples following different incubation times were observed under a JEOL JSM-6610LV microscope (JEOL Ltd., Tokyo, Japan) according to the method by Haga and Ohashi [15]. Pork loin samples were placed on scanning electron microscopy (SEM) stubs and sputter-coated with gold. Images were acquired at an accelerating voltage of 10 kV.

### Antioxidant activity of pork protein hydrolysate hydrolyzed by kiwifruit juice

To prepare the samples for antioxidant evaluation, pork loins injected with kiwifruit juice and incubated for 0 and 24 h were extracted as described by Zhang et al [16]. The crude protein content of the extract was adjusted to 0.3 mg/mL and stored at −20°C for 24 h prior to antioxidant analysis by the 2,2-diphenyl-1-picrylhydrazyl (DPPH) radical scavenging assay and reducing power assay according to the methods described by Huang et al [17].

The DPPH radical scavenging activity of hydrolysate extracted from pork loin was evaluated by the decrease in the absorbance of the solution at 517 nm, and the result was measured as a percentage obtained from the following equation:


[(1-(absorbance of the sample/absorbance of the sample)]×100.

The reducing power was evaluated by determining the absorbance at 700 nm. Results were expressed as absorbance, and a higher absorbance indicated a higher reducing power.

### Statistical analyses

The whole experiments were replicated three times (n = 3). Data were analyzed either by one-way analysis of variance (ANOVA) or two-way ANOVA using SPSS 20.0 statistical software (SPSS, Inc., Chicago, IL, USA). The results were presented as mean±standard error of the mean. The factors were treatments and incubation times (0, 4, 8, and 24 h) and their interactions, and to find out the effect of both factors, the means were compared (significance level set at 5%) using Duncan’s multiple range test.

## RESULTS AND DISCUSSION

### Protease activities of the green and gold kiwifruit juices

We determined that GRJ (38.05±1.08) had a higher protease activity than GOJ (30.13±2.95) (p<0.05). This result was consistent with the previous study that green kiwifruit had a higher specific protease activity than gold kiwifruit [18]. Chao [19] reported that actinidin content determined protease activity even among kiwifruit species with similar protein concentrations. Thus, the difference in specific protease activity of GRJ and GOJ in this study is considered the effect of actinidine content.

### Physicochemical properties of the pork loin injected with green and gold kiwifruit juice

#### pH and color values of pork loin before cooking

The addition of kiwifruit juice decreased the pH values of pork loin and this was dose dependent, regardless of the type of kiwifruit juice (p<0.05; Table 2). pH decrease of pork loin injected with kiwifruit juice might affect the enzyme in kiwifruit juice on the ionic strength of the meat. Since pH values of GRJ and GOJ used in this study were 3.3 and 3.2, respectively, the acidity of the kiwifruit juice may affect the pH values of pork loin depending on the injection amount. These results are consistent with previous study as reported by Liu et al [20]. However, no changes in pH depending were observed during incubation time in our study (p>0.05).

Since the interaction between treatment (type and level of kiwifruit) and incubation time had no significant effect on the color (L*, a*, b*) of pork loin before cooking, data were pooled by treatment within incubation time and the incubation time within the treatments (p>0.05; Table 2). L* and b* values differed among the treatments (p<0.05). Control (CTL, without kiwifruit juice) showed the lowest L* among the treatments (p<0.05), confirming that the application of kiwifruit juice into pork tended to be brighter. No differences in L* value among the type of kiwifruit juice at the same injection level were observed (p>0.05). As we expected, the more GOJ injected level into the pork loin, the higher value the L* value (p<0.05). The b* value of pork loin injected with 20% kiwifruit juice, regardless of the type of kiwifruits, GOJ or GRJ, was higher than that of the CTL (p>0.05). When 10% kiwifruit juice was injected into pork loin, the b* value was the similar to those of the CTL (p>0.05). Pork loin injected with 20% GOJ showed the highest b* value among the treatments (p<0.05), because, in green kiwifruit, the kiwifruit flesh appears green due to chlorophyll, whereas the flesh color of gold kiwifruit is yellow due to the effect of β-carotene and lutein, which are contained in gold kiwifruit [21]. It is considered that the color difference between pork loin treated with GRJ and GOJ was due to the difference in the original pigment composition and color characteristics between the gold and green kiwifruit.

#### pH and color values of pork loin after cooking

Although no differences in the pH values of pork loin during the incubation time were observed after cooking (p>0.05), there was a difference among the treatments (p<0.05; Table 2). The pH values of pork loin treated with kiwifruit juice were lower than those of the CTL, and the pH values decreased with increasing level of kiwifruit juice injected into pork loin, regardless of the type of kiwifruit juice (p<0.05). Zhang et al [16] reported that moisture loss from the reaction of the enzyme applied to meat products might affect the pH reduction of meat. Leygonie et al [22] noted that the decrease in the pH of meat caused the increase of the solute concentration and the release of hydrogen ions due to the loss of fluid from the meat tissue. Based on these results, water loss during the cooking process of pork loin treated with kiwifruit juice caused a decrease in pH values in our study.

The color values of pork loin treated with kiwifruit juice are presented in Table 2. There were no differences among the treatments (p>0.05) which indicated that the type of kiwifruit juice and injection dose did not cause color alteration in cooked pork loin. Moreover, the color values showed no difference during the incubation time (p>0.05). The color values of cooked meat might be due to the combination of the maillard reaction and myoglobin denaturation, which produced compounds that contribute to the final brown color of the cooked meat [23]. Our study confirmed that the color of pork loins was affected by the browning that occurs when meat is cooked rather than by the color of the kiwifruit juice applied to the meat.

#### Cooking loss

There was no difference in the CL by type of kiwifruit juice (p>0.05), and the CL was higher than that of the CTL when 20% kiwifruit juice was applied into pork loins (p<0.05; Table 2). CL increased with increasing levels of GRJ injected (p<0.05). However, no further increases between 10% GOJ and 20% GOJ were observed (p>0.05). There was no difference in CL between 0 and 8 h of the incubation time, but it increased at 24 h. The increase in CL of meat products treated with actinidin from different kiwifruit cultivars has been discussed in a previous study [9]. The high CL in the 20% kiwifruit juice treatment might affect extensive myosin fragmentation. Myosin maintains the integrity of the myofibrils, a major structural component responsible for water binding and entrapment within the muscle fibers [20]. Moreover, Purslow et al [24] noted that disruption of muscle structure during aging and an increase in small peptide fragments of sarcoplasmic proteins and cytoskeleton in meat decreased the water holding capacity and increased CL in meat. Thus, we suggested that the destruction of the muscle structure following treatment with 20% GRJ observed in our study allowed water to escape from the structure more easily than when GRJ was applied at the lower addition level (10%).

#### Warner–Bratzler shear force value

**Figure 1:**
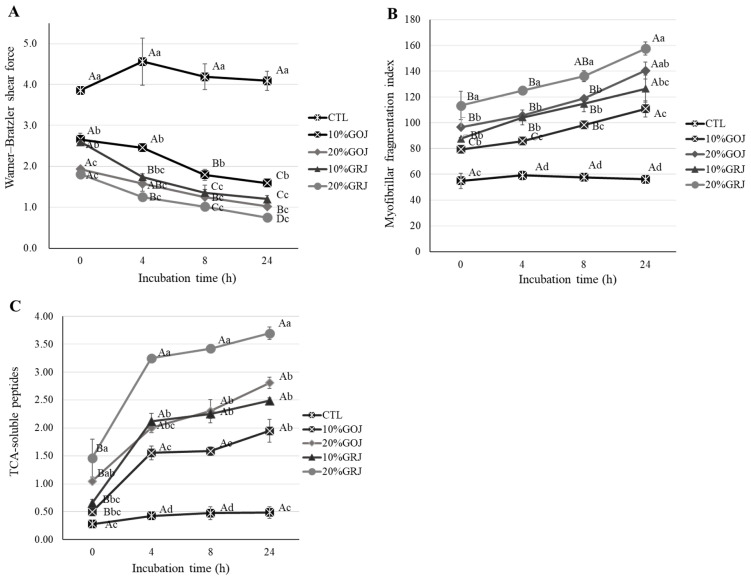
WBSF of pork loin injected with gold and green kiwifruit juice (GOJ and GRJ) and incubated at 4°C for 0, 4, 8, and 24 h (A), MFI of pork loin injected with GOJ and GRJ and incubated at 4°C for 0, 4, 8, and 24 h (B) and trichloroacetic acid (TCA)-soluble peptides of pork loin injected with GOJ and GRJ and incubated at 4°C for 0, 4, 8, and 24 h (C). WBSF, Warner–Bratzler shear force; MFI, myofibril fragmentation index; GOJ, gold kiwifruit juice GRJ, green kiwifruit juice; CTL, pork loins injected to 110% of their original weight with brine solution; 10% GOJ/20% GOJ, pork loins injected to 110% of their original weight with brine solution containing 10% and 20% GOJ; 10% GRJ/20% GRJ, pork loins injected to 110% of their original weight with brine solution containing 10% and 20% GRJ. ^A–D^ Means with different superscripts in the same treatment are significantly different (p<0.05; Duncan test). ^a–d^ Means with different superscripts in the same incubation time are significantly different (p<0.05; Duncan test).

In Figure 1A, all treatments with kiwifruit juice showed lower WBSF compared to the CTL (p<0.05). When a 20% level of kiwifruit juice was applied for 24 h incubation, the same WBSF was shown, regardless of the type of kiwifruit juice (p>0.05), however in 10% of cases, GRJ showed a lower WBSF than GOJ from 4 h incubation (p<0.05). GOJ showed a difference at various addition level for all incubation times, whereas GRJ showed a difference at the injection dose only between at 0 and 24 h incubation (p<0.05). At 24 h incubation, 10% GRJ showed the same WBSF as 20% GOJ (p>0.05), and GRJ showed higher tenderness effect than GOJ. Regardless of the application level, there was no difference in the WBSF of GOJ after 8 h incubation (p>0.05). The WBSF of GRJ-treated pork loin decreased at 8 and 24 h incubation for 10% GRJ and 20% GRJ, respectively. The reduction in WBSF of the pork loin treated with kiwifruit was explained previously [3,25] by the tenderizing effect of proteolytic enzymes contained in kiwifruit. Based on this, it is judged that the WBSF of pork loin was reduced by the action of actinidin contained in GOJ and GRJ. Gong et al [7] reported that green kiwifruit extracts had higher myofibrillar proteolytic activity than gold kiwifruit extract, which is similar to the results of our study. In our study, the relatively higher proteolytic activity of GRJ suggested that more protein cleavage could be induced due to the influence of its endopeptidase activity. This, in turn, explained the higher tenderness induced by GRJ than GOJ when applied to meat.

#### Myofibril fragmentation index

The MFI indicates myofibrillar protein degradation and meat tenderness [26]. Figure 1B shows the effects of kiwifruit juice on the MFI of kiwifruit juice injected pork loin as compared to those without kiwifruit juice (CTL). As compare to the control, marked increase in MFI was observed in the kiwifruit juice-treated pork loin. Comparing the type of kiwifruit juice, GRJ showed a higher MFI (p<0.05), and 10% GRJ and 20% GOJ showed the similar MFI value until 8 h incubation (p>0.05). All treatments increased throughout incubation, and the kiwifruit-treated pork loin samples showed the highest MFI value at 24 h as compared to other incubation times. A 10% GRJ injected pork loin didn’t not differ MFI values between the 8 and 24 h incubation times (p>0.05), indicating that the incubation time could be shortened. The MFI indicates both the breakage of the I-band and the cleavage of the inter-myofibril connection [27]. Actinidin is one of several exogenous proteases in kiwifruit that can contribute to meat tenderness [5]. In our study, the increase in MFI of pork loin treated with kiwifruit juice might be related to the degradation of myofibrillar proteins into segments at or near the Z-disk during incubation. In addition, GRJ-treated pork loins showed a higher MFI value than the GOJ-treated pork loins, which indicates that GRJ might contains more actinidin level as compared to GOJ.

#### Trichloroacetic acid-soluble peptides

The TCA-soluble peptide is related to the index for muscle protein degradation and tenderness [2]. The changes in TCA-soluble peptides in pork loin injected with kiwifruit juice during 24 h of refrigerated storage are shown in Figure 1C. We observed that 10% GOJ and 20% GRJ had the lowest and highest content of TCA-soluble peptides, respectively, during incubation (p< 0.05), although 10% GRJ was the same as 20% GOJ at 4 and 8 h incubation (p>0.05). Regardless of the type of kiwifruit, pork loin injected with kiwifruit juice showed more TCA-soluble peptides compared to the CTL (p<0.05). This result was supported by the previous study of Zhao et al [28] who reported that enzymatic treatment of intact proteins increased protein solubility by degrading them into peptides and amino acids. In our study, the degradation of meat protein by the enzymes in kiwifruit juice resulted in the release of peptides, and the proteolytic activity of the enzymes even occurred during refrigerated storage. GRJ produced more TCA-soluble peptides compared to GOJ, suggesting that the enzymatic content and activity of GRJ were suitable for the proteolysis of meat products. The TCA-soluble peptides increased during 24 h of incubation for samples treated with kiwifruit juice. In particular, the TCA-soluble peptide increased rapidly between 0 and 4 h incubation. Ha et al [6] reported that kiwifruit-derived actinidin hydrolyzed myofibrils at a slower rate, as compared to the other plant enzymes, and hydrolysis appeared after 2 h. Considering these results, GRJ treatment progressively increased the TCA-soluble peptides from 4 h incubation.

#### Sodium dodecyl sulfate-polyacrylamide gel electrophoresis

**Figure 2:**
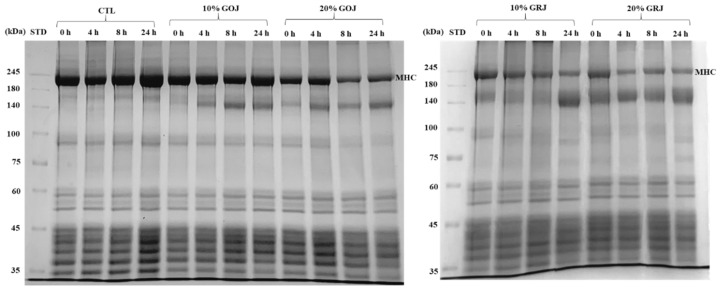
Pork loin protein hydrolyzing activity of gold and green kiwifruit juice analyzed with sodium dodecyl sulfate–polyacrylamide gel electrophoresis patterns. GOJ, gold kiwifruit juice GRJ, green kiwifruit juice; CTL, pork loins injected to 110% of their original weight with brine solution; 10% GOJ/20% GOJ, pork loins injected to 110% of their original weight with brine solution containing 10% and 20% GOJ; 10% GRJ/20% GRJ, pork loins injected to 110% of their original weight with brine solution containing 10% and 20% GRJ.

The profiles of injected pork loin as affected by different type and levels of kiwifruit juice during incubation are illustrated in Figure 2. For the incubation time, the band distribution and intensity changes of the meat protein samples injected with kiwifruit juice differed from the CTL. Upon examining the SDS-PAGE gel pattern, more protein hydrolysis of meat proteins occurred in pork loin treated with kiwifruit juice, especially GRJ, compared to the CTL. According to the type of kiwifruit juice at 20%, the band intensity for pretreatment with GRJ decreased faster with increased incubation time. A similar trend was found at 10%; moreover, 10% GOJ did not differ from CTL. For example, among meat proteins to which kiwifruit juice was applied except for 10% GOJ, the band intensity of the myosin heavy chain, approximately 35, 37, and 55 kDa decreased. In particular, 20% GRJ showed a tendency to weaken the band strength from 4 h incubation, showing faster degradation than 10% GRJ and 20% GOJ. The incubation time and band intensity differed for each sample. In particular, the band corresponding to 70 kDa appeared only in pork loin samples treated with GRJ. Considering the band intensity during incubation time, GRJ might have a higher protein degradation ability than GOJ. Consistent with our results, Gong et al [7] reported that green kiwifruit extract was more effective in promoting the hydrolysis of protein from pork loins as compared to gold kiwifruit extract. Furthermore, the results of myofibrillar degradation using the kiwifruit juice were consistent with the results of Ha et al [6], who found that GRJ hydrolyzed not only filamin, desmin, and actin, but also high molecular weight proteins such as nebulin and titin, and hydrolysis of myofibrillar protein occurred 5 min after incubation with GRJ.

#### Scanning electron microscopy

**Figure 3:**
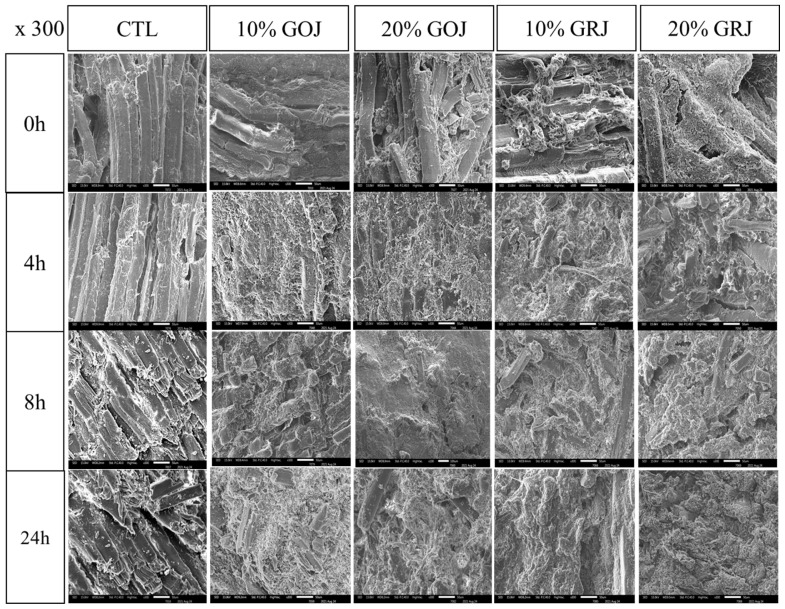
Scanning electron microscopy (SEM) images of pork loin treated with gold and green kiwifruit juice at ×300 magnification. GOJ, gold kiwifruit juice GRJ, green kiwifruit juice; CTL, pork loins injected to 110% of their original weight with brine solution; 10% GOJ/20% GOJ, pork loins injected to 110% of their original weight with brine solution containing 10% and 20% GOJ; 10% GRJ/20% GRJ, pork loins injected to 110% of their original weight with brine solution containing 10% and 20% GRJ.

The SEM images in Figure 3 demonstrate the effect of the type and injection level of kiwifruit juice on the microstructural changes of pork loins. CTL showed organized myofibrils and sarcomere tissue bundles throughout incubation, and the fibrous structures were complete, clear, and tightly connected. By contrast, cracks appeared across the lengths of the muscle fibers in pork loin treated with kiwifruit juice, especially at the higher injection level (20%). Compared with GOJ, GRJ caused more dramatic changes, including gaps and obvious cracks among the muscle fibers, destruction of the integrity of muscle fibers and the fiber layer, and fragmentation of the myofibril structure, irrespective of the injection level. Both 10% and 20% GOJ displayed that muscle fiber dissolution and fracture occurred in the area with wide muscle fibers from 4 and 24 h, respectively. Muscle fiber decomposition was faster in pork loin to which GRJ was applied than GOJ at the same level from 4 h for 10% GRJ and 0 h for 20% GRJ. This phenomenon is partially due to protein breakdown by the action of actinidin in kiwifruit juice. Zhu et al [4] reported that the application of actinidin to beef caused structural changes in myofibrils around the Z-discs, suggesting protein hydrolysis. Overall, the SEM images confirmed that the muscle fiber destruction observed after kiwifruit juice injection was consistent with all microscopic tissue parameters, indicating an increased effect on muscle tenderness and weakening of the microstructure.

##### Antioxidant activity

The antioxidant activities of the pork loin meat samples that were hydrolyzed with two types and levels of kiwifruit juice were measured by the DPPH scavenging activity and reducing power assay (Table 3). During incubation for up to 24 h, 20% GRJ showed the highest antioxidant activity among the other treatments (p<0.05). The 10% GOJ, 20% GOJ, and 10% GRJ treatments showed the similar antioxidant activity at 0 h incubation (p>0.05). Interestingly, the GRJ-treated samples showed higher antioxidant activity than the GOJ-treated samples at the same level of addition (p<0.05). It is suggested that GRJ promoted more meat hydrolysis than GOJ, forming peptides with more antioxidant activity.

For the comparison of the antioxidant activity with various incubation time, the antioxidant activity values of 20% GOJ, 10% GRJ, and 20% GRJ increased with incubation time (p<0.05). It was confirmed that the number of antioxidant peptides increased with protein degradation of kiwifruit juice-treated pork loin during incubation. Teh et al [29] reported that the more activated the protease, the greater the proteolysis and the higher the bioactivity, as the DPPH scavenging activity of the hydrolysates is generally related to the proteolytic activity. Yu et al [30] stated that changes in the number of peptides produced by the hydrolysis of proteins could cause to increase antioxidant activity. Moreover, they found that some hydrophobic peptides generated through microbial enzymes incubated on pork protein had antioxidant activity. Based on this, it was confirmed that hydrolysis of pork loins by kiwifruit juice protease not only increased the generation of free peptides with antioxidant capacity, but also enriched the antioxidant amino acids during the incubation time.

## CONCLUSION

GRJ had a similar tenderizing ability and muscle fiber degrading effect to GOJ, even when a 10% injection was applied to pork loin. Although 20% GRJ injection improved the tenderness of pork loin better than 10% GRJ injection, there had problems related to the increased CL. Except for 10% GOJ, pork loin treated with kiwifruit juice increased its antioxidant activity as incubation progressed. In addition, 20% GRJ showed the highest activity throughout incubation, and 10% GRJ showed the similar antioxidant activity as 20% GOJ. These results indicated that GRJ could be used as an effective tenderizing agent to improve the eating properties like softening even when a small amount is applied to meat compared to GOJ. Moreover, it is a potential method to enrich the peptides associated with antioxidant properties that could act as natural preservatives in the muscle food system in the future.

## Figures and Tables

**Table 1:** Formulation of chicken breast with different colored kiwifruit juice

Item	Treatment^1)^				

	CTL	10% GOJ	20% GOJ	10% GRJ	20% GRJ
Ingredient (%)					
Water	81.7	71.7	61.7	71.7	61.7
Salt	8.0	8.0	8.0	8.0	8.0
Sugar	8.0	8.0	8.0	8.0	8.0
Sodium tripolyphosphate	2.3	2.3	2.3	2.3	2.3
GOJ	-	10	20	-	-
GRJ	-	-	-	10	20
Total	100	100	100	100	100

GOJ, gold kiwifruit juice GRJ, green kiwifruit juice.

1) CTL, pork loins injected to 110% of their original weight with brine solution; 10% GOJ/20% GOJ, pork loins injected to 110% of their original weight with brine solution containing 10% and 20% GOJ; 10% GRJ/20% GRJ, pork loins injected to 110% of their original weight with brine solution containing 10% and 20% GRJ.

**Table 2:** Effect of gold and green kiwifruit juice and incubation time on physicochemical properties of pork loin before and after cooking

Items	Before cooking				After cooking				
	
	pH	L*	a*	b*	pH	L*	a*	b*	Cooking loss
Treatment^1)^									
CTL	5.70±0.03^a^	46.34±1.65^d^	1.16±0.86^a^	5.01±1.27^c^	6.01±0.05^a^	72.34±2.27^a^	2.50±1.45^a^	9.45±1.47^a^	20.24±3.57^b^
10% GOJ	5.69±0.04^b^	55.02±2.16^c^	0.66±1.64^a^	6.75±2.02^bc^	5.90±0.13^ab^	72.32±1.52^a^	2.14±0.64^a^	9.41±1.72^a^	23.57±4.71^ab^
20% GOJ	5.42±0.10^c^	58.21±3.30^a^	0.66±1.38^a^	7.20±1.82^b^	5.70±0.16^c^	74.39±1.69^a^	2.58±1.53^a^	9.92±0.94^a^	27.91±5.83^a^
10% GRJ	5.55±0.15^b^	53.32±3.68^bc^	0.23±1.16^a^	6.46±0.76^bc^	5.84±0.11^b^	73.18±1.86^a^	2.94±1.70^a^	9.85±1.25^a^	22.15±1.94^b^
20% GRJ	5.41±0.03^c^	57.35±3.05^ba^	0.45±0.92^a^	9.58±1.98^a^	5.65±0.14^c^	71.50±3.09^a^	2.77±1.02^a^	9.93±2.00^a^	27.84±3.86^a^
Incubation time (h)									
0	5.56±0.16^a^	52.25±3.97^a^	1.48±1.61^a^	6.99±1.51^a^	5.92±0.17^a^	73.62±2.93^a^	2.33±0.49^a^	9.85±1.27^a^	21.51±3.23^b^
4	5.61±0.17^a^	54.74±4.35^a^	0.11±0.86^a^	7.07±1.73^a^	5.84±0.18^a^	72.91±1.69^a^	2.39±1.17^a^	9.52±1.44^a^	24.12±3.96^ab^
8	5.55±0.17^a^	54.78±6.23^a^	0.30±0.89^a^	6.91±2.65^a^	5.82±0.14^a^	72.41±2.36^a^	3.15±1.65^a^	9.94±1.90^a^	24.77±5.07^ab^
24	5.56±0.14^a^	54.42±5.73^a^	0.63±0.97^a^	7.04±2.85^a^	5.77±0.19^a^	71.93±1.91^a^	2.47±1.54^a^	9.53±1.33^a^	27.08±6.37^a^

GOJ, gold kiwifruit juice; GRJ, green kiwifruit juice.

1) CTL, pork loins injected to 110% of their original weight with brine solution; 10% GOJ/20% GOJ, pork loins injected to 110% of their original weight with brine solution containing 10% and 20% GOJ; 10% GRJ/20% GRJ, pork loins injected to 110% of their original weight with brine solution containing 10% and 20% GRJ.

a–d For each attribute, means within a column with different letters are significantly different (p<0.05; Duncan test).

**Table 3:** DPPH radical scavenging activity and reducing power of hydrolysate from pork loin proteins by gold and green kiwifruit juices

Treatment^1)^	DPPH radical scavenging (%)		Reducing power (optical density)	
	
	0 h	24 h	0 h	24 h
CTL	12.77±6.72^cA^	15.70±6.06^dA^	0.34±0.05^cA^	0.34±0.04^dA^
10% GOJ	34.24±3.89^bA^	35.32±3.27^cA^	0.48±0.03^bA^	0.50±0.04^cA^
20% GOJ	40.38±7.58^bB^	53.01±5.50^bA^	0.50±0.08^bB^	0.61±0.06^bA^
10% GRJ	36.21±5.18^bB^	46.15±4.41^bA^	0.49±0.05^bB^	0.56±0.06^bA^
20% GRJ	52.74±9.09^aB^	62.39±6.63^aA^	0.58±0.03^aB^	0.75±0.06^aA^

DPPH, 2,2-diphenyl-1-picrylhydrazyl; GOJ, gold kiwifruit juice GRJ, green kiwifruit juice.

1) CTL, pork loins injected to 110% of their original weight with brine solution; 10% GOJ/20% GOJ, pork loins injected to 110% of their original weight with brine solution containing 10% and 20% GOJ; 10% GRJ/20% GRJ, pork loins injected to 110% of their original weight with brine solution containing 10% and 20% GRJ.

a–d Means with different superscripts in the same incubation time are significantly different (p<0.05; Duncan test).

A,B Means with different superscripts in the same treatment are significantly different (p<0.05; Duncan test).
